# Whole genome sequence of the emerging oomycete pathogen *Pythium insidiosum* strain CDC-B5653 isolated from an infected human in the USA

**DOI:** 10.1016/j.gdata.2015.11.019

**Published:** 2015-11-23

**Authors:** Marina S. Ascunce, Jose C. Huguet-Tapia, Edward L. Braun, Almudena Ortiz-Urquiza, Nemat O. Keyhani, Erica M. Goss

**Affiliations:** a Emerging Pathogens Institute, University of Florida, Gainesville, FL, United States; b Department of Plant Pathology, University of Florida, Gainesville, FL, United States; c Department of Biology and Genetics Institute, University of Florida, Gainesville, FL, United States; d Department of Microbiology and Cell Science, University of Florida, Gainesville, FL, United States

**Keywords:** Oomycete, *Pythium insidiosum*, Pythiosis, Human emerging pathogen, Genome sequencing

## Abstract

*Pythium insidiosum* ATCC 200269 strain CDC-B5653, an isolate from necrotizing lesions on the mouth and eye of a 2-year-old boy in Memphis, Tennessee, USA, was sequenced using a combination of Illumina MiSeq (300 bp paired-end, 14 millions reads) and PacBio (10  Kb fragment library, 356,001 reads). The sequencing data were assembled using SPAdes version 3.1.0, yielding a total genome size of 45.6 Mb contained in 8992 contigs, N_50_ of 13 Kb, 57% G + C content, and 17,867 putative protein-coding genes. This Whole Genome Shotgun project has been deposited at DDBJ/EMBL/GenBank under the accession JRHR00000000.

**Table:** 

Specifications	
Organism/cell line/tissue	*Pythium insidiosum* strain CDC-B5653
Sex	Not applicable
Sequencer or array type	Illumina MiSeq and PacBio
Data format	Assembled
Experimental factors	CDC sample originally isolated from necrotizing lesions on the mouth and eye of a 2-year-old boy
Experimental features	Whole genome shotgun sequencing followed by genome assembly and gene description
Consent	Not applicable
Sample source location	ATCC 200269

## 1 Direct link to deposited data

http://www.ncbi.nlm.nih.gov/nuccore/JRHR00000000.1.

## 2 Experimental design, materials and methods

The oomycete genus *Pythium* comprises more than 250 described species [1], most of which are saprobes or facultative plant pathogens that cause seed rot and damping-off, root, stem and fruit rot, foliar blight, and postharvest decay [2]. *P. insidiosum* is the only *Pythium* species that causes disease in mammals. It is the causal agent of pythiosis, a deadly disease of horses, dogs, and other mammals in tropical and subtropical regions [3], [4]. Pythiosis also affects humans, and was first reported in Thailand in 1985 [5], [6].

Whole genome sequencing was applied to *P. insidiosum* ATCC 200269 strain CDC-B5653, which was originally isolated from necrotizing lesions on the mouth and eye of a 2-year-old boy in Memphis, Tennessee, USA. A combination of Illumina MiSeq (300 bp paired-end, 14 millions reads) and PacBio (10 Kb fragment library, 356,001 reads) sequencing data were used to assemble the genome using SPAdes version 3.1.0 [7], yielding a total size of 45.6 Mb contained in 8992 contigs, N_50_ of 13 Kb, maximum contig length of 148 Kb, and 57% G + C content. We used Augustus version 3.0.1 [8] to predict genes *ab initio*, using a gene model previously described for *Pythium* [9]. This genome contains 225 tRNA and 17,867 putative protein-coding genes. To create a representative set of orthologous groups for *P. insidiosum* and its closest relatives, genomes from the following seven *Pythium* species were included: *P. ultimum var. ultimum*, *P. arrhenomanes*, *P. irregulare*, *P. aphanidermatum*, *P. iwayamai*, *P. ultimum var. sporangiiferum*, and *P. vexans* (now *Phytopythium vexans* [10]). These genomes were downloaded from the *Pythium* Genome Database (http://pythium.plantbiology.msu.edu/download.shtml) [9], [11]. Reciprocal BLAST analysis on all genomes indicated that *P. insidiosum* shares 5922 unique orthologous genes with the other *Pythium* genomes and has 649 taxon specific genes. *P. insidiosum* shares more orthologs (233) with *P. aphanidermatum* and *P. arrhenomanes* than with the remaining *Pythium* species. These findings indicate that the three species are evolutionarily close to each other, which is consistent with estimates of *Pythium* phylogeny based on ITS sequences [1]. Further analysis will examine genes and gene families that distinguish the *P. insidiosum* genome from those of plant other pathogenic oomycetes.
